# The Effect of Angiotensin II, Retinoic Acid, EGCG, and Vitamin C on the Cardiomyogenic Differentiation Induction of Human Amniotic Fluid-Derived Mesenchymal Stem Cells

**DOI:** 10.3390/ijms21228752

**Published:** 2020-11-19

**Authors:** Monika Gasiūnienė, Elvina Valatkaitė, Aistė Navakauskaitė, Rūta Navakauskienė

**Affiliations:** 1 Life Sciences Center, Department of Molecular Cell Biology, Institute of Biochemistry, Vilnius University, Saulėtekio av. 7, LT-10257 Vilnius, Lithuania; monika.gasiuniene@gmail.com (M.G.); elvina.valatkaite@gf.stud.vu.lt (E.V.); 2 Faculty of Medicine, Vilnius University, Čiurlionio st., 21, LT-03101 Vilnius, Lithuania; aiste.navakauskaite@mf.stud.vu.lt

**Keywords:** amniotic fluid, stem cells, cardiac, differentiation, chromatin, metabolism

## Abstract

Human amniotic fluid-derived mesenchymal stem cells (AF-MSCs) may be potentially applied in cell therapy or regenerative medicine as a new alternative source of stem cells. They could be particularly valuable in restoring cardiac tissue after myocardial infarction or other cardiovascular diseases. We investigated the potential of biologically active compounds, namely, angiotensin II, retinoic acid (RA), epigallocatechin-3-gallate (EGCG), vitamin C alone, and the combinations of RA, EGCG, and vitamin C with angiotensin II to induce cardiomyogenic differentiation of AF-MSCs. We observed that the upregulated expression of cardiac gene markers (NKX2-5, MYH6, TNNT2, and DES) and cardiac ion channel genes (sodium, calcium, the potassium) as well as the increased levels of Connexin 43 and Nkx2.5 proteins. Extracellular flux analysis, applied for the first time on AF-MSCs induced with biologically active compounds, revealed the switch in AF-MSCs energetic phenotype and enhanced utilization of oxidative phosphorylation for energy production. Moreover, we demonstrated changes in epigenetic marks associated with transcriptionally active (H3K4me3, H3K9ac, and H4hyperAc) or repressed (H3K27me3) chromatin. All in all, we demonstrated that explored biomolecules were able to induce alterations in AF-MSCs at the phenotypic, genetic, protein, metabolic, and epigenetic levels, leading to the formation of cardiomyocyte progenitors that may become functional heart cells in vitro or in vivo.

## 1. Introduction

Myocardial infarction and other cardiovascular diseases are some of the main causes of morbidity and mortality across the world. The biggest issue is the irreversible damage of the cardiac tissue due to heart failure and its low regenerative capacity. Conventional treatment (drugs or operations) is expensive and usually not effective enough; thus, there is a huge demand for alternative therapies, such as cardiac tissue restoration using stem cells and regenerative medicine. One of the alternative sources of stem cells for heart tissue engineering is human amniotic fluid-derived mesenchymal stem cells (AF-MSCs). AF-MSCs express pluripotency gene markers, such as OCT4, SOX2, NANOG, and REX1, as seen for embryonic stem (ES) cells, and they are also positive for mesenchymal stem cells surface markers, for example, CD44, CD90, CD105, and others [1,2]. Moreover, AF-MSCs have a wide differentiation potential toward cell lineages of all three germ layers, for example, endoderm (hepatocytes), mesoderm (osteocytes, myocytes, adipocytes, and chondrocytes), and ectoderm (neurons) [3,4]. Thus, these stem cells occupy the niche between pluripotent embryonic and multipotent adult stem cells [5] and may be potentially utilized in cell therapy.

We and others have previously shown that AF-MSCs are capable of differentiating into cardiomyocyte precursors. One of the widely applied strategies is to use chemical agents, such as DNA methyltransferase (DNMT) inhibitors decitabine (5-aza-2′-deoxycytidine) and zebularine [6,7] or p53 inhibitor pifithrin-α [7] as cardiomyogenic differentiation inducers. However, they may be toxic to the organism and cause undesirable side effects [8]. Because of that, there is a growing interest in the search for natural, safe, and efficient inducers of cardiac differentiation. In our previous work, we have demonstrated that angiotensin II (AngII) and transforming growth factor β1 (TGF-β1), natural biomolecules found in various human tissues, are able to induce genetic, metabolic, and epigenetic alterations in AF-MSCs leading to the initiation of cardiomyogenic differentiation [9]. These natural differentiation inducers are not the only substances able to initiate cardiac differentiation; all-*trans* retinoic acid (RA) was demonstrated as an efficient agent causing cardiomyogenic differentiation of mouse embryonic stem (ES) cells [10,11]. In addition, vitamin C, also known as ascorbic acid, was used to generate beating cardiomyocytes from mouse ES cells [12,13,14,15] or from induced pluripotent stem cells [16] and even for transdifferentiation of mouse fibroblasts to cardiomyocytes [17]. We also tested epigallocatechin-3-gallate (EGCG), a polyphenol extracted from green tea, which is a widely used antioxidant having DNMT and histone deacetylase (HDAC) inhibitory properties [18]. EGCG was demonstrated to have antiadipogenic features and potential for obesity treatment [19], as well as anticancer, anti-inflammatory, anticollagenase, antifibrosis, and osteogenesis promotion effects in cancer and stem cells [20,21,22]. Furthermore, EGCG was successfully applied after myocardial infarction and reduced the infarct volume and size, as well as inhibited cardiac myocyte apoptosis and oxidative stress [23,24,25,26] and even enhanced adipose tissue MSC differentiation into endothelial progenitor cells [27], suggesting its potential as a cardiac differentiation inducer. Thus, we aimed to observe the effect of these agents and their combinations with angiotensin II on cardiomyogenic differentiation induction of human AF-MSCs and to evaluate the processes in the cells during the induced differentiation.

This study was designed to evaluate the ability of natural compounds, namely, retinoic acid (RA), vitamin C, and EGCG, alone or in combination with angiotensin II to induce cardiomyogenic differentiation of human AF-MSCs. We explored morphology, as well as alterations in the expression of cardiomyocytes gene markers and cardiac ion channel genes, during the induced differentiation, and we determined the levels and localization of Nkx2.5 and Connexin 43 proteins showing successful initiation of cardiac differentiation. For the first time, cellular flux analysis was applied to AF-MSCs differentiated with RA, vitamin C, and EGCG alone or together with AngII, revealing the switch in cell energy phenotype from glycolysis to oxidative phosphorylation. We also studied epigenetic changes, i.e., modified histones, in the AF-MSCs differentiated toward the cardiomyogenic lineage that indicated a global chromatin changeover accompanying other processes in the cells.

## 2. Results

### 2.1. Human AF-MSC Characterization

**Figure 1:**
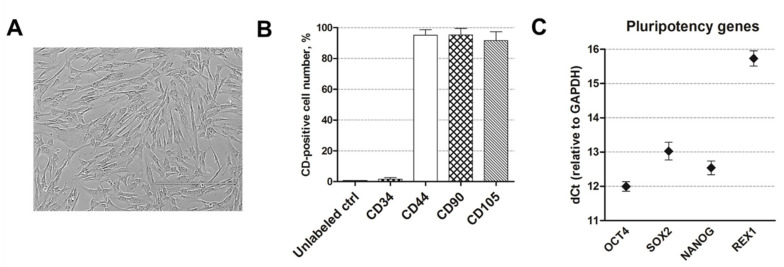
Human amniotic fluid-derived mesenchymal stem cells (AF-MSCs) characterization. (**A**) The typical morphology of human amniotic fluid-derived mesenchymal stem cells, grown in cell culture. Scale bar = 400 µm. (**B**) The expression of the main cell surface markers CD44, CD90, CD105, and CD34 as detected by flow cytometry. Unlabeled ctrl: unlabeled, undifferentiated control cells. Results are presented as the mean ± SD (*n* = 3). (**C**) The relative expression of pluripotency gene markers, namely, OCT4, SOX2, NANOG, and REX1, as determined by RT-qPCR. Data, relative to GAPDH, are presented as the mean ± SD (*n* = 3).

Human amniotic fluid-derived mesenchymal stem cells, used in this study, were isolated from the second-trimester amniocentesis samples of a healthy pregnancy and possessed a typical spindle-shaped morphology (Figure 1A). More than 95% of isolated AF-MSCs expressed mesenchymal cell surface markers, namely, CD44 (cell adhesion molecule), CD90 (Thy-1, thymocyte antigen-1), and CD105 (endoglin), and less than 1% expressed hematopoietic cell marker CD34 (Figure 1B) as measured using flow cytometry. Undifferentiated AF-MSCs were also positive for pluripotency gene markers, namely, OCT4, SOX2, NANOG, and REX1, as detected by RT-qPCR (Figure 1C). 

### 2.2. Assessment of Cardiac Differentiation Initiation in AF-MSCs

**Figure 2:**
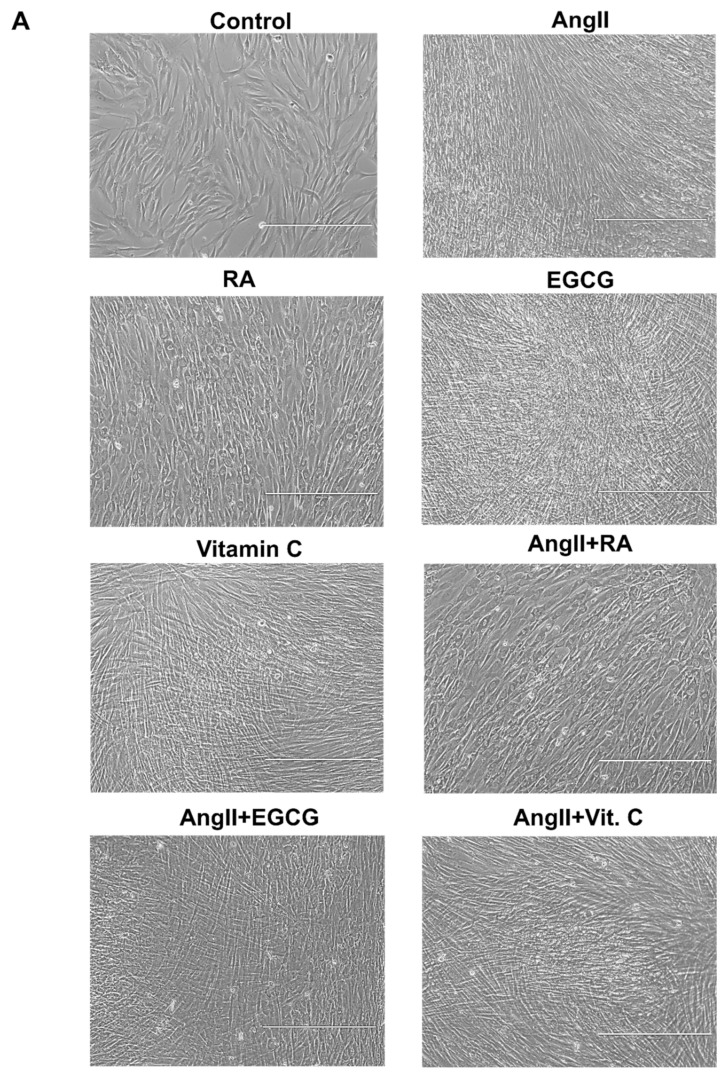

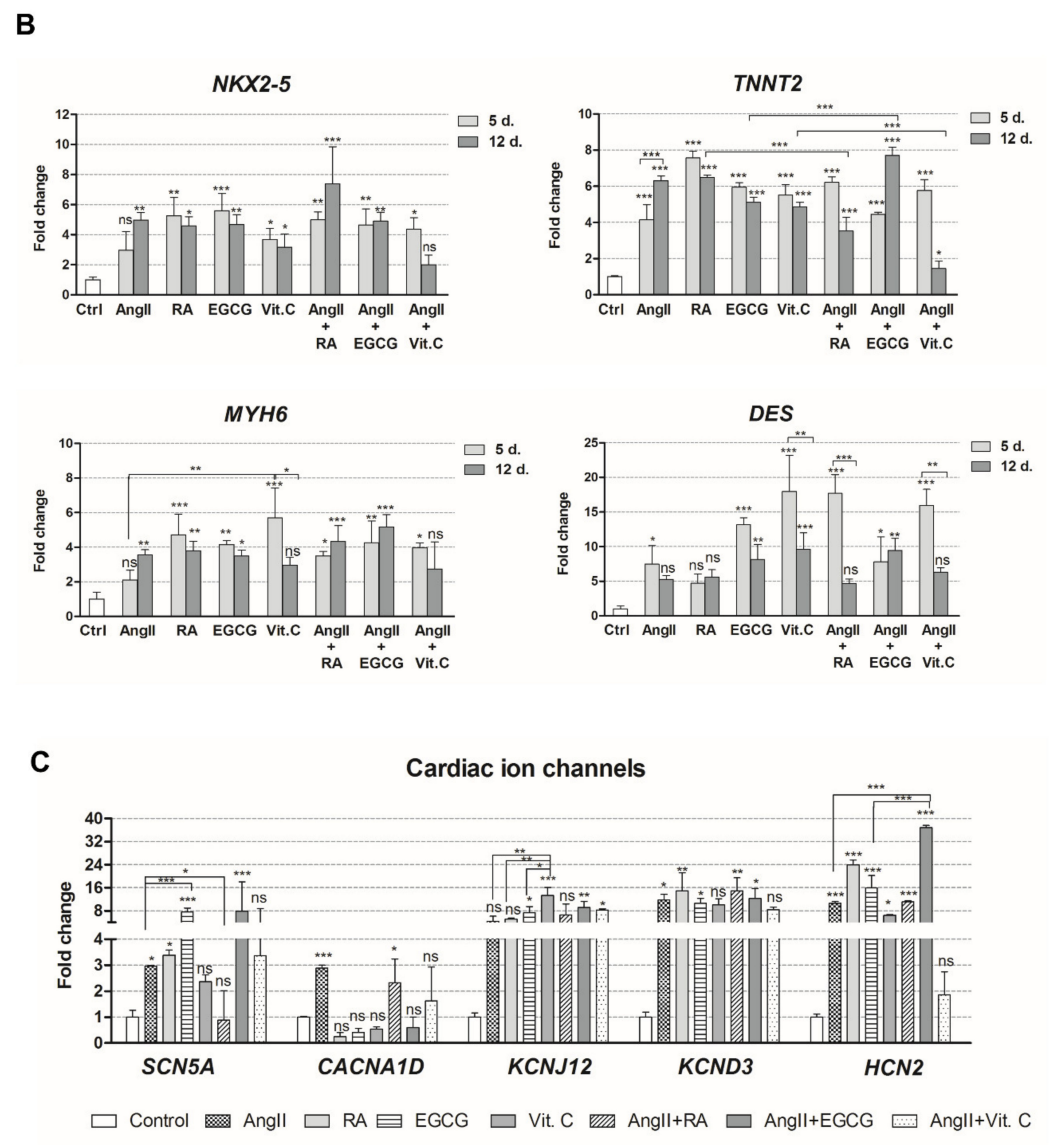
Assessment of cardiac differentiation of AF-MSCs at the morphological and gene expression levels. (**A**) Morphological alterations of AF-MSCs, induced to differentiate into cardiac lineage using angiotensin II (AngII), retinoic acid (RA), epigallocatechin gallate (EGCG), vitamin C, and their combinations, on the 12th day after the initiation of differentiation. Scale bar = 400 µm. (**B**) The relative expression of the main cardiac gene markers NKX2-5, MYH6, TNNT2, and DES on days 5 (5 d.) and 12 (12 d.) after induced differentiation. Ctrl: nondifferentiated control cells. (**C**) The relative expression of cardiac ion channels genes on day 12 after differentiation: SCN5A (sodium voltage-gated channel α-subunit 5), CACNA1D (L-type calcium channel), KCNJ12 and KCND3 (voltage-gated potassium), and HCN2 (hyperpolarization-activated cyclic nucleotide-gated channel). The relative gene expression was determined by RT-qPCR, normalized to GAPDH, and presented as a fold change over the undifferentiated control. The data are presented as the mean ± SD (*n* = 3); *p* ≤ 0.05 (*), *p* ≤ 0.01 (**), *p* ≤ 0.001 (***), ns: nonsignificant.

Cardiomyogenic differentiation of AF-MSCs was induced using different biologically active compounds or their combinations: angiotensin II (AngII), retinoic acid (RA), epigallocatechin gallate (EGCG), vitamin C, angiotensin II together with retinoic acid (AngII + RA), angiotensin II with EGCG (AngII + EGCG), and angiotensin II with vitamin C (AngII + Vit. C). Firstly, the morphological alterations compared to undifferentiated cells were assessed 12 days after the induction of cardiac differentiation. AF-MSCs, when treated with all agents except RA, became elongated, formed a tight monolayer, and started forming junctions between adjacent cells (Figure 2A). Interestingly, the addition of retinoic acid to the differentiation medium made AF-MSCs bigger and round-shaped with clearly visible nuclei. This effect was also detectable in AngII + RA-induced cells. Next, the induction of cardiomyogenic differentiation was evaluated at the gene expression level. The relative expression of the main cardiac gene markers NKX2-5 (encoding an early cardiac transcription factor Nkx2.5), MYH6 (α-myosin heavy chain), TNNT2 (cardiac troponin T), and DES (Desmin) was upregulated in AF-MSCs differentiated with all agents or their combinations at days 5 and 12 of differentiation compared to undifferentiated control (Figure 2B). The effect of RA, EGCG, and vitamin C here was at a comparable level to angiotensin II; moreover, their combinations with AngII did not induce much higher expression of the studied genes in comparison to angiotensin II or the applied agents alone. In addition, the relative expression of several cardiac ion channels genes was detected: SCN5A—sodium voltage-gated channel α-subunit 5, CACNA1D—L-type calcium channel, KCNJ12—the ATP-sensitive inward rectifier potassium channel, KCND3—the transient outward potassium channel, and HCN2—the hyperpolarization-activated cyclic nucleotide-gated channel (Figure 2C). AngII increased the expression of almost all tested genes, while vitamin C induced only KCNJ12 and HCN2 expression. Furthermore, the AngII + Vit. C combination performed the worst in upregulating cardiac ion channel genes. EGCG upregulated the SCN5A gene the most, while its combination with AngII caused the highest increase in HCN2 expression. Surprisingly, the expression of CACNA1D was enhanced only in AngII- and AngII + RA-induced AF-MSCs. All in all, these gene expression patterns indicated the initiation of cardiac-like phenotype formation.

**Figure 3:**
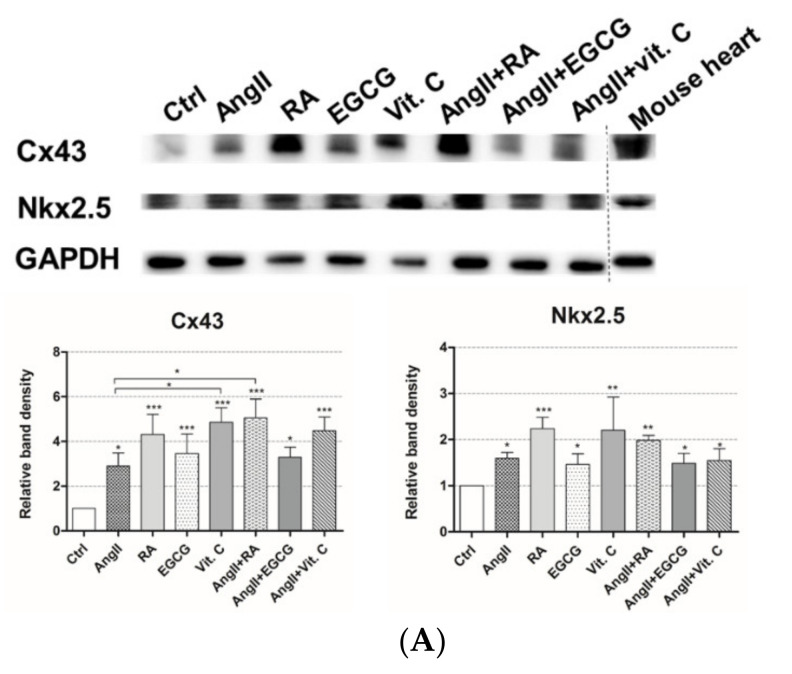

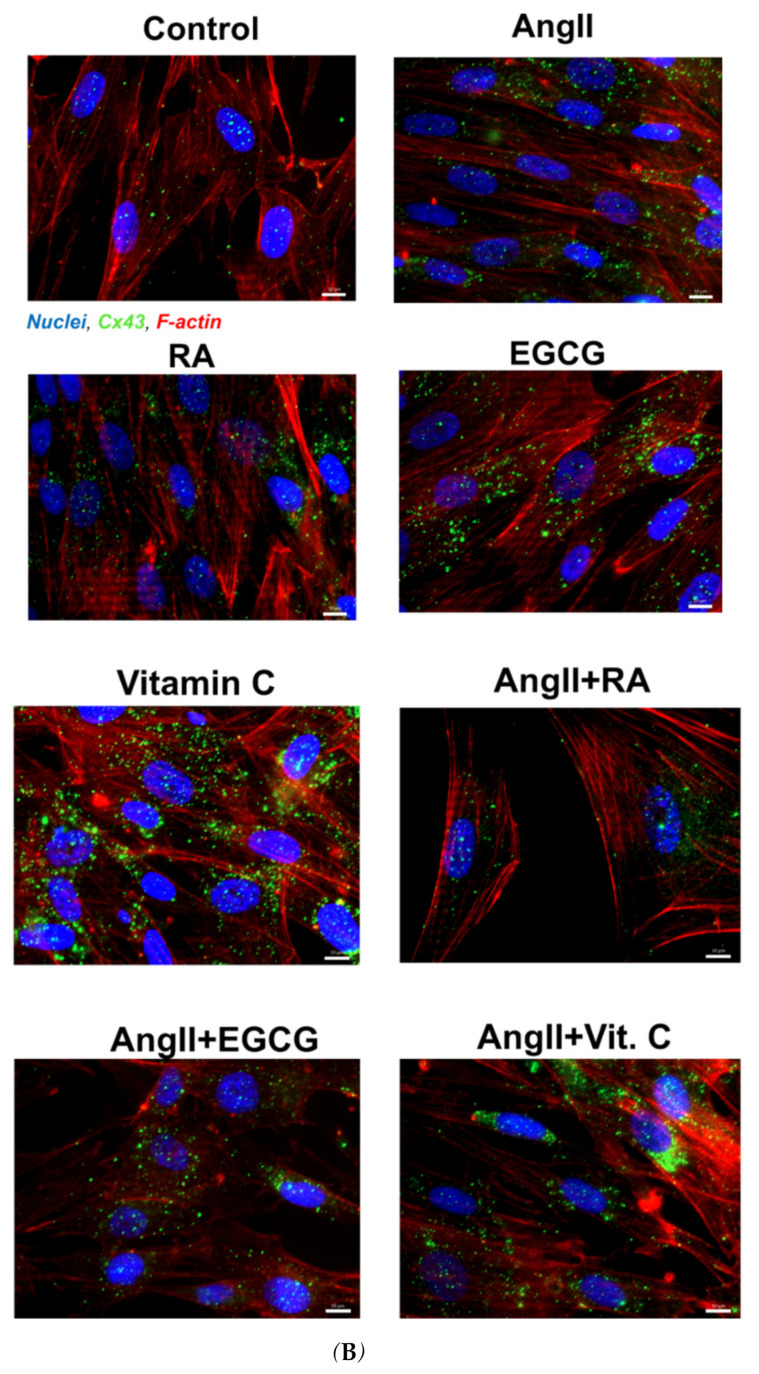

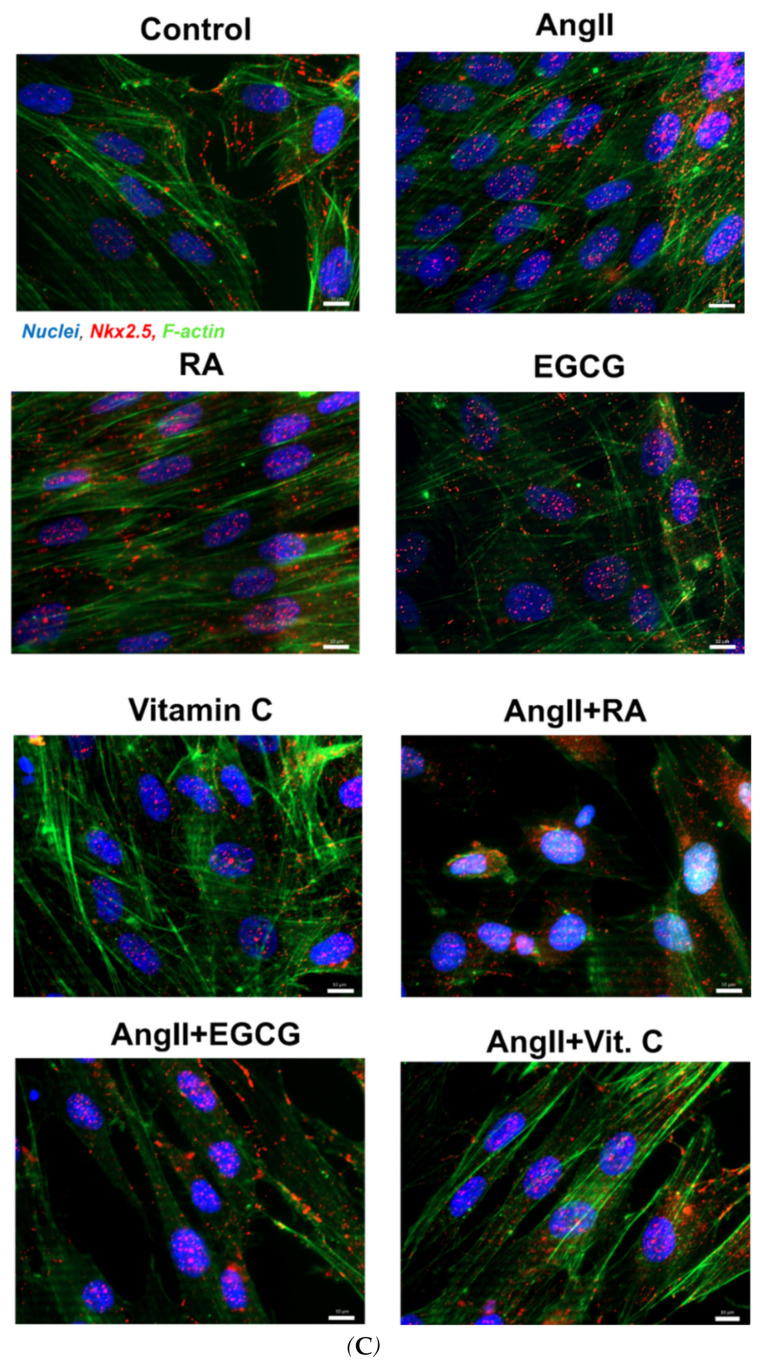

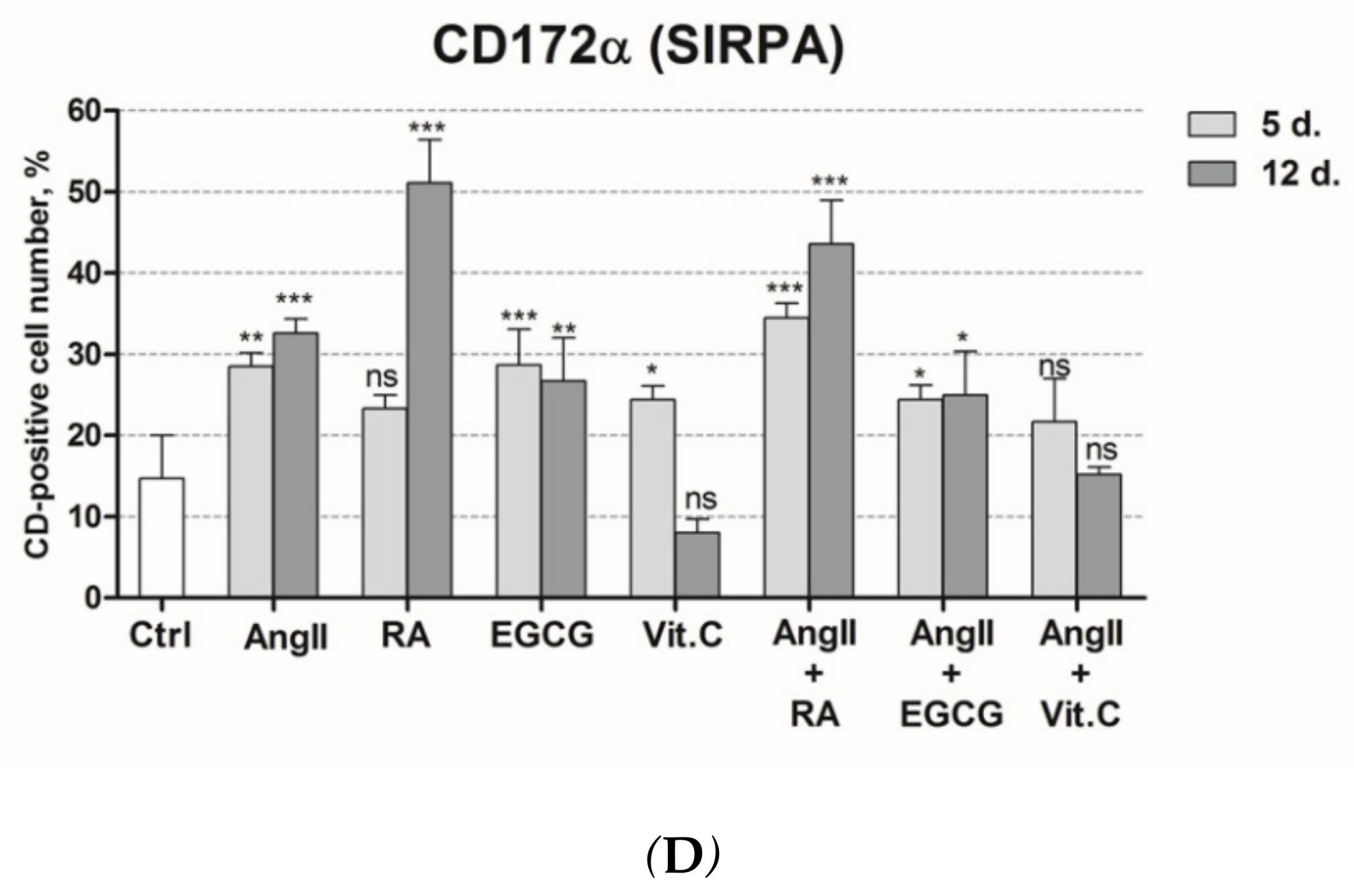
Evaluation of induced AF-MSCs differentiation at the protein level. (**A**) The levels of the main gap junction protein, Connexin 43 (Cx43), and the early cardiac transcription factor Nkx2.5, as determined by Western blot in nondifferentiated cells (Ctrl) and differentiated cells on day 12 after differentiation. Mouse heart lysate was used as a positive control. The relative density of each band was measured using ImageJ software (NIH, USA), normalized to the GAPDH loading control, and presented as a fold difference over control. The data are presented as the mean ± SD (*n* = 3); *p* ≤ 0.05 (*), *p* ≤ 0.01 (**), *p* ≤ 0.001 (***), ns: nonsignificant. The blots represent one of three independent experiments showing similar results. (**B**) The distribution of Cx43 in control and induced AF-MSCs on day 12 after differentiation obtained using the immunofluorescence assay. Nuclei are stained blue, Cx43 is stained green, and F-actin is stained red. Samples were analyzed using a Zeiss Axio Observer fluorescence microscope, with a 63× objective in immersion oil. Scale bar = 10 µm. (**C**) The localization of Nkx2.5 protein in control and induced cells on day 12 after differentiation. Nuclei are stained blue, Nkx2.5 is stained red, and F-actin is stained green. Samples were analyzed using a Zeiss Axio Observer fluorescence microscope, with a 63× objective in immersion oil. Scale bar = 10 µm. (**D**) The expression of cardiomyocyte-specific cell surface marker CD172α (SIRPA) in control AF-MSCs (Ctrl) and AF-MSCs induced to differentiation on days 5 (5 d.) and 12 (12 d.) after differentiation as determined by flow cytometry. The data are presented as the mean ± SD (*n* = 3); *p* ≤ 0.05 (*), *p* ≤ 0.01 (**), *p* ≤ 0.001 (***), ns: nonsignificant.

This was also demonstrated at the protein level. The levels of Connexin 43 (Cx43) protein, the main component of cardiac gap junctions, were enhanced in AF-MSCs, induced with all differentiation agents, on the 12th day after differentiation in agreement with the high levels of Cx43 in adult mouse heart (Figure 3A). The highest increase in Cx43 levels was detected in RA-, vitamin C-, AngII + RA-, and AngII + Vit. C-induced cells. Analysis of Cx43 levels and localization using immunofluorescence matched the Western blot results, showing the increased amount of this protein in induced cells (Figure 3B). The majority of Cx43 was diffused throughout the cells; on the other hand, a minor fraction of Cx43 that was distributed at the boundaries of AF-MSCs may indicate the beginning of the formation of gap junctions. Moreover, Nkx2.5, the early cardiac transcription factor, was evaluated not only at the gene but also at the protein level. As Western blot results uncovered, the levels of Nkx2.5 were upregulated in AF-MSCs differentiated with all biomolecules or their combinations, especially in RA-treated cells concomitant to a high amount of Nkx2.5 in the mouse heart (Figure 3A). Immunofluorescence data complemented Western blot results, demonstrating that the amount of Nkx2.5 was enhanced, and it was mostly localized in the nuclei of differentiated cells (Figure 3C). 

Furthermore, we evaluated the expression of cardiomyocyte surface marker CD172α (SIRPA) on AF-MSCs differentiated toward the cardiac lineage on days 5 and 12 (Figure 3D). As flow cytometry data revealed, CD172α expression increased on all induced AF-MSCs compared to undifferentiated control cells. On AngII-, RA-, and AngII + RA-treated cells, the levels of CD172α gradually raised from the beginning of differentiation until day 12. AF-MSCs, induced with EGCG and AngII + EGCG, maintained similar levels of this surface marker during differentiation, while, on AF-MSCs induced with vitamin C and AngII + Vit. C, the expression of CD172α decreased as differentiation proceeded. Nevertheless, successful initiation of cardiac differentiation of AF-MSCs was also confirmed at the protein level.

### 2.3. Cellular Energetics and Metabolic Alterations during the Induced Differentiation

**Figure 4:**
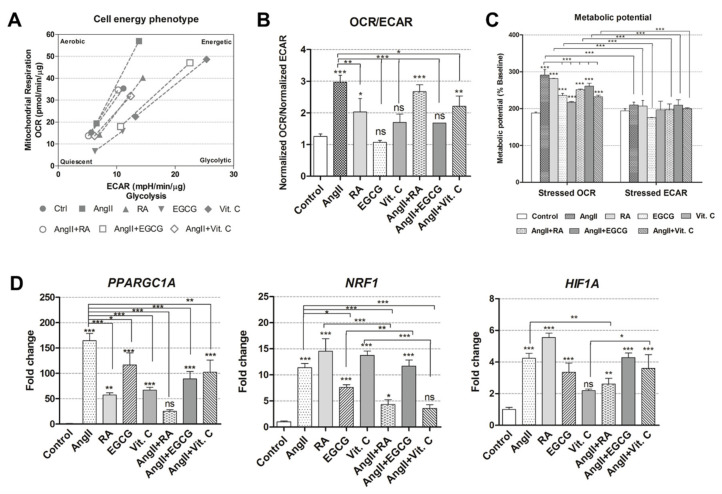
Cell energy phenotype and metabolic alterations during the induced differentiation of AF-MSCs. (**A**) Cell energy diagram representing energy phenotype shift in basal (lower points) and stressed (higher points) conditions. Oxygen consumption rate (OCR) and extracellular acidification rate (ECAR) of control and differentiated cells (12 days) were normalized to the total amount of protein. (**B**) The ratio of normalized OCR to normalized ECAR in control and induced AF-MSCs. (**C**) Metabolic potential (as a percentage) of undifferentiated and induced AF-MSCs was calculated from stressed OCR/ECAR over baseline OCR/ECAR. (**D**) The relative expression of genes related to cell metabolism and respiration: PPARGC1A (peroxisome proliferator-activated receptor gamma coactivator 1 alpha), NRF1 (nuclear respiratory factor 1), and HIF1A (hypoxia-inducible factor 1-alpha), in control and differentiated cells as determined using RT-qPCR. The data were normalized to GAPDH and are presented as a fold change over undifferentiated control. Results in (**B**–**D**) are presented as the mean ± SD (*n* = 3); *p* ≤ 0.05 (*), *p* ≤ 0.01 (**), *p* ≤ 0.001 (***), ns: nonsignificant.

After the demonstration of cardiomyogenic differentiation initiation at various levels (morphological, transcriptional, and translational), we aimed to uncover alterations in cellular energetics and metabolism. The Seahorse extracellular flux analyzer was used to measure these parameters in undifferentiated control and induced AF-MSCs on day 12. Firstly, the basal oxygen consumption rate (OCR) and the basal extracellular acidification rate (ECAR) were measured simultaneously. Then, stressed conditions were induced by adding the inhibitors of the electron transfer chain (carbonyl cyanide-4 (trifluoromethoxy) phenylhydrazone (FCCP) and oligomycin); then, OCR and ECAR were measured again. The obtained results indicated that the phenotype of AF-MSCs, induced with AngII, RA, AngII + EGCG, and vitamin C, switched toward a more energetic one (Figure 4A). On the other hand, EGCG-, AngII + RA-, and AngII + Vit. C-treated AF-MSCs remained at the undifferentiated control level. In addition, the basal OCR/ECAR ratio revealed that AngII-, RA-, AngII + RA-, and AngII + Vit. C-differentiated AF-MSCs started utilizing mitochondrial oxidative phosphorylation (OXPHOS) more than glycolysis (Figure 4B) because of the higher OCR/ECAR ratio, where more energy is generated through OXPHOS. In AF-MSCs, induced with vitamin C and AngII + EGCG, OCR/ECAR ratio was lower compared to other induced cells but slightly higher than control cells, while EGCG-treated AF-MSCs used both OXPHOS and glycolysis at a similar level to the undifferentiated control. Under high energy demand (induced stress conditions), all differentiated AF-MSCs had a higher-stressed OCR compared to the uninduced control, as well as stressed ECAR, suggesting that they relied more on mitochondrial respiration (Figure 4C).

Together with alterations in metabolism and energetics, the expression of several genes, associated with metabolism regulation, was measured using RT-qPCR (Figure 4D). PPARGC1A (peroxisome proliferator-activated receptor gamma coactivator 1 alpha) was upregulated in all induced cells except for AngII + RA, whereas angiotensin II caused the highest increase in expression. All differentiation inducers except for AngII + Vit. C caused an increase in the expression of NRF1 (nuclear respiratory factor 1), and HIF1A (hypoxia-inducible factor 1-alpha) was upregulated in AF-MSCs treated with all agents except vitamin C.

### 2.4. Epigenetic Changes during the Induced Differentiation

**Figure 5:**
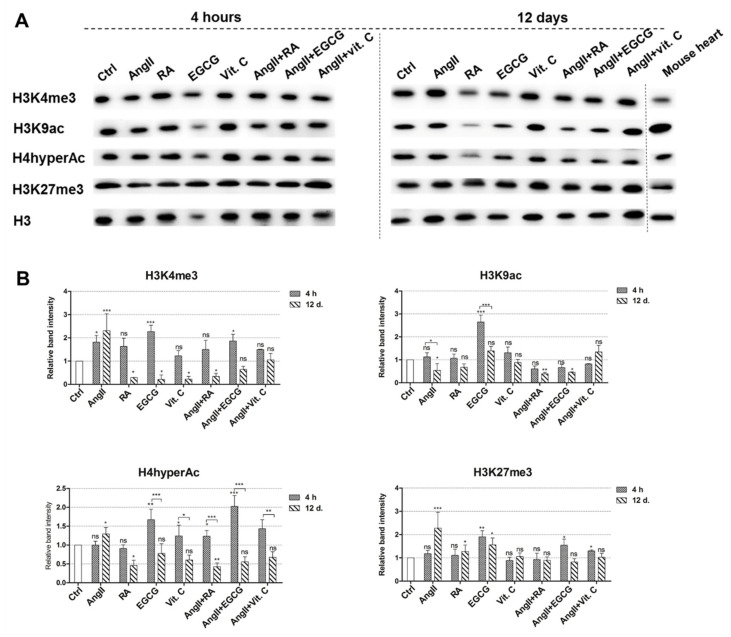
Epigenetic alterations during the induced cardiomyogenic differentiation. (**A**) Total proteins were extracted from control cells (Ctrl) and cells induced using AngII, RA, EGCG, vitamin C, and their combinations, 4 h and 12 days after the induction of differentiation. Alterations in the levels of modified histones considered as markers of an active chromatin state (H3K4me3, H3K9ac, and H4hyperAc) and markers of a repressed chromatin state (H3K27me3) are presented as determined by Western blot. Mouse heart lysate was used as a positive control. The blots represent one of three independent experiments showing similar results. (**B**) The relative band density of modified histones measured using ImageJ software (NIH, USA) and normalized to the H3 loading control. The data are presented as the mean ± SD (*n* = 3); *p* ≤ 0.05 (*), *p* ≤ 0.01 (**), *p* ≤ 0.001 (***), ns: nonsignificant.

Epigenetic changes, i.e., alterations in histone modification, were studied using immunoanalysis (Figure 5A). As changes in histone modifications are very dynamic, we approached their levels at the very beginning of differentiation, 4 h after the induction of differentiation, and at a later stage after 12 days. At the initiation of differentiation, the levels of modified histones associated with a transcriptionally active chromatin state, i.e., H3K4me3, H3K9ac, and H4hyperAc, were upregulated in AF-MSCs, induced with almost all agents (Figure 5B). The H3K4me3 histone marker was upregulated the most AF-MSCs, treated with AngII, EGCG, and AngII + EGCG. EGCG and its combination with angiotensin II caused the highest increase in hyperacetylated H4 levels together with vitamin C and AngII + Vit. C. Furthermore, AF-MSCs differentiated using EGCG had enhanced levels of another acetylated histone—H3K9ac. On the other hand, the histone marker related to a transcriptionally repressed or paused chromatin state in bivalent domains, H3K27me3, remained at a similar level to that seen in control undifferentiated AF-MSCs 4 h after differentiation.

Interestingly, the levels of histone modifications of active chromatin decreased on day 12 after induced differentiation. H3K4me3 levels on day 12 were only higher than after 4 h in AngII-differentiated cells; in all other cells, this histone marker diminished and its levels were smaller compared to the undifferentiated control and to the levels after 4 h of differentiation. Both acetylations (H3K9ac and H4hyperAc) were also reduced in AF-MSCs treated with all agents. However, H3K27me3, a marker of repressed chromatin, increased in AngII-, RA-, and EGCG-induced AF-MSCs or remained at the control level in vitamin C-, AngII + RA-, AngII + EGCG-, and AngII + Vit. C-treated cells. Together, these alterations in the levels of modified histones indicated a global chromatin changeover during the induced cardiomyogenic differentiation, from a more transcriptionally open and active state at the beginning of differentiation to a more compact state at the end of differentiation.

## 3. Discussion

Stem cells and especially mesenchymal stem cells (MSCs) are being used more and more in the field of cell therapy or regenerative medicine for the restoration of damaged heart tissue. One of the most common sources of MSCs, widely studied in vitro, in vivo, and in clinical trials, is bone marrow MSCs [28]. However, they limitations. The amount of MSCs in bone marrow is very minor [29]; thus, they need to be cultivated in vitro to obtain the number of cells sufficient for clinical utilization and enter senescence during long-term cultivation. Moreover, their stemness and differentiation potential decrease [30,31,32]. In this context, amniotic fluid mesenchymal stem cells emerge as a safe and potential alternative to bone marrow MSCs possessing all the features of MSCs as proposed by the International Society for Cellular Therapy [33], as well as having additional advantages, such as wide differentiation potential not only into mesoderm but also into endoderm or ectoderm lineages [3,4]. Moreover, AF-MSCs have longer in vitro cultivation capacity while maintaining their characteristics and enter senescence much later than bone marrow MSCs [32]. These features make them potentially useful in cell therapy or regenerative medicine. 

In this study, we sought to explore the potential of AF-MSCs to differentiate into cardiomyocyte progenitors using several natural biologically active compounds, namely, angiotensin II, retinoic acid, EGCG, vitamin C, and their combinations. We previously demonstrated that angiotensin II is able to induce alterations in human AF-MSCs at various levels (genetic, metabolic, and epigenetic) leading to the initiation of cardiomyogenic differentiation [9]. As our results demonstrated for the first time, RA, EGCG, and vitamin C, as well as their combinations with angiotensin II, were also able to initiate cardiac differentiation of AF-MSCs, albeit to a different extent. Our results are in agreement with Wobus and colleagues [10] showing that retinoic acid influenced the differentiation efficiency of mouse ES cells into the cardiomyogenic lineage. Furthermore, RA treatment specifically increased the number of ventricle cardiomyocytes [11]. Interestingly, the morphology of our RA-treated AF-MSCs differed the most from other induced cells, possibly due to the different nature of cardiomyocyte progenitors. Vitamin C also caused the upregulation of cardiac-specific genes (transcription factor NKX2-5), cardiac muscle-specific genes (MYH6, TNNT2, and DES), and cardiac ion channel genes (SCN5A, CACNA1D, KCNJ12, KCND3, and HCN2), consistent with previous studies using stem cells from several different sources [12,16,34]. Vitamin C was also demonstrated as the enhancer of the yield of beating cardiomyocytes [14,15,16,17], but we did not detect any spontaneous contractions in our induced AF-MSCs. Surprisingly, EGCG, the compound with a huge action potential for different treatments [20], was not previously tested as a direct cardiomyogenic differentiation inducer. However, EGCG was successfully applied in vitro as an inducer of endothelial differentiation of adipose tissue MSCs [27] or in vivo for cardiomyopathy or infarct treatment and enhanced cardiac function restoration [35], where it reduced the infarct volume [25,26] or decreased the infarct size and inhibited cardiac myocyte apoptosis [23,24]. As our results indicated, EGCG was equally efficient to other tested agents at the gene expression level during the induced differentiation. In addition, in most cases, we did not detect any additional effect using combinations of RA, EGCG, and vitamin C with angiotensin II compared to angiotensin II or these agents alone. 

All of these agents also caused an increase in Connexin 43 levels in induced AF-MSCs according to our results. Connexin 43 is known as the main component of gap junctions necessary for mediating electrical and chemical coupling between cardiomyocytes [36], observed at the boundaries of the cells. Despite the fact that, in our induced cells, Cx43 was spread throughout the whole cell, we noticed a tendency of its accumulation at the cell periphery, suggesting that intercalated discs, where Cx43 forms gap junctions and sustains electrical activity for myocardial function, started forming. We also obtained that Nkx2.5, the early cardiac transcription factor, responsible for the transcription of structural and functional cardiac genes, was also upregulated in all induced AF-MSCs and localized in the nuclei, proving its importance in cardiac differentiation [37]. For the first time, we tested the expression of a cell surface marker CD172α (SIRPA) on AF-MSCs induced to cardiomyogenic differentiation and detected up to 40–50% of positive AF-MSCs (induced using RA and AngII + RA). This cell surface marker is usually found on cardiac progenitors or contracting cardiomyocytes obtained from ES cells [38,39]. Recently, CD172α expression was reported on human umbilical cord MSCs with pericyte properties [40] and adipose tissue MSCs [41] differentiated toward cardiac lineage. We demonstrated that it could also be detected on differentiated human AF-MSCs. 

At the gene and protein level, all tested differentiation inducers had a rather similar effect; only RA and vitamin C worked better in some cases. However, more differences appeared from the cell energy phenotype data showing the different nature of applied inducers and the possible maturation level of AF-MSCs after the induction of cardiac differentiation. The obtained results indicated that, after treatment with angiotensin II, RA, AngII + EGCG, and vitamin C, the AF-MSC phenotype shifted toward a more energetic one, while it remained at the undifferentiated control level in AF-MSCs differentiated with other agents. These results are in agreement with Capasso et al. [42] showing that undifferentiated MSCs may use the tricarboxylic acid cycle or anaerobic glycolysis for ATP production, while differentiated cells or specialized progenitors begin to use oxidative phosphorylation more and more [43]. Consistent with previous research demonstrating that human induced pluripotent stem cell-generated cardiomyocytes applied mostly oxidative phosphorylation to meet the enhanced energy demand [44,45], our AF-MSCs, induced using all tested agents, used mitochondrial respiration more than glycolysis under maximum energy demand. Moreover, genes related to metabolism regulation (PPARGC1A, NRF1, and HIF1A) were upregulated in almost all differentiated cells, but to a different extent. The greatest increase in relative expression was observed for the PPARGC1A gene, which is enriched in highly oxidatively active tissues, for example, the heart, and which is activated under enhanced energy demand [46]. PPARGC1A also promotes mitochondrial proliferation and activates nuclear respiratory factors 1 and 2 (NRF1/2) [47]. NRF1 is vital for mitochondrial biogenesis and respiration in the heart tissue [48], while HIF1A regulates the adaptive response to hypoxia and is related to cardiac differentiation stimulation in ES cells [49]. Considering these cellular energetics and metabolism results together with the upregulation of cardiomyocyte-specific and metabolic genes and proteins, angiotensin II-, RA-, and vitamin C-differentiated cells showed a commitment toward cardiomyocytes. Meanwhile, AF-MSCs, treated with EGCG or combinations of differentiation inducers, demonstrated an enhanced use of oxidative phosphorylation only under induced stress conditions, suggesting the less mature state of cardiomyocytes progenitors.

Our studied epigenetic changes occurring during cardiac differentiation and controlling the fate of stem cells involved modified histones associated with a transcriptionally open or repressed chromatin state. Despite slight variations in the levels of modified histones, we explored a tendency; at the very beginning of the differentiation (4 h from the induction), global levels of active chromatin histone modifications H3K4me3, H3K9ac, and H4hyperAc increased, and the level of repressed DNA mark H3K27me3 remained similar to that in the undifferentiated control cells, contrary to the end of differentiation (12 days), when the levels of active chromatin modifications decreased and that of H3K27me3 increased. These epigenetic alterations may contribute to the determination of differentiation level of stem cells, as Sdek and colleagues stated that high acetylation and methylation of H3 and H4 suggest an immature cardiac phenotype, while, in more differentiated cardiac progenitor cells, histone acetylation decreases and methylation related to transcriptional suppression (H3K9me3 and H3K27me3) predominates [50]. In addition, Wang et al. demonstrated that promoters of cardiac-specific genes, such as MYH6, TNNT2, and others, are enriched in higher levels of acetylated histones H3 and H4 [51]. Furthermore, it was shown that the reduced expression of histone deacetylases 1 and 2 was related to the increased expression of structural and functional genes of cardiomyocytes [52]. All in all, such alterations in histone modifications levels during the induced cardiac differentiation reflect a global chromatin changeover from the open state, where differentiation-related genes are gradually activated and pluripotency-maintaining genes are gradually repressed, to a more closed and less active state, keeping a more differentiated status of AF-MSCs.

In conclusion, human AF-MSCs could be induced toward a cardiomyogenic lineage in vitro using natural, bioactive compounds, such as angiotensin II, retinoic acid, and vitamin C, while EGCG had a considerably smaller effect. Moreover, RA, EGCG, and vitamin C combinations with angiotensin II did not improve differentiation efficiency in most cases. We demonstrated that these biomolecules caused alterations in the gene and protein expression patterns, as well as metabolic and epigenetic landscape, leading to cardiomyocytes progenitor formation. The differentiated AF-MSCs could be considered as being at the commitment stage, i.e., at the onset of induced differentiation with an immature phenotype, with the potential to become functional beating heart muscle cells under specific conditions in vitro or in vivo. Therefore, our results expand the knowledge about human amniotic fluid mesenchymal stem cells and provide additional insights into the molecular processes occurring at the initiation of induced cardiomyogenic differentiation. However, more research is needed before testing the potential of differentiated AF-MSCs in cell therapy for the treatment of cardiac illnesses. 

## 4. Materials and Methods 

### 4.1. Isolation and Cultivation of Human Amniotic Fluid Mesenchymal Stem Cells

Second-trimester amniotic fluid (about 5 mL) containing amniotic fluid mesenchymal stem cells was obtained from healthy women (age 28–30, gestational week 16–17) who needed prenatal diagnostics but where no genetic abnormalities were detected (protocols approved by the Ethics Committee of Biomedical Research of Vilnius District, No 158200-123-428-122). Stem cells were isolated using a two-stage protocol as described in our previous work [2] and maintained in the growth medium DMEM (4.5 g/L glucose) supplemented with 10% FBS, 100 U/mL penicillin, and 100 µg/mL streptomycin (Gibco, Thermo Fisher Scientific, New York, NY, USA).

### 4.2. Cell Surface Marker Analysis Using Flow Cytometry

Cell surface markers of undifferentiated AF-MSCs were detected using the protocol from Glemžaitė and Navakauskienė [53] with fluorescein isothiocyanate (FITC)-conjugated mouse anti-human antibodies against CD44 (cat. no. 156-3C11; Invitrogen, Thermo Fisher Scientific, Carlsbad, CA, USA), CD34 (cat. no. 130-113-178; Miltenyi Biotec, Bergisch Gladbach, Germany), and CD90 (cat. no. 11-0909-42; Molecular Probes, Thermo Fisher Scientific, Hillsboro, OR, USA), PE-conjugated mouse anti-human against CD105 (cat. no. 12-1057-42; Invitrogen, Thermo Fisher Scientific, Carlsbad, CA, USA), and mouse IgG2A-FITC (cat. no. 130-113-833) Miltenyi Biotec, Bergisch Gladbach, Germany), IgG1-FITC (cat. no. RMG101), IgG2b-FITC (cat. no. IgG2b-FITC) (Invitrogen, Thermo Fisher Scientific, Carlsbad, CA, USA), or IgG1-PE (cat. no. GM4993) (Molecular Probes, Thermo Fisher Scientific, Hillsboro, OR, USA) as isotype controls. The cell surface marker of differentiated AF-MSCs, CD172α (SIRPA, cat. no. 15-414), was detected using the same protocol and allophycocyanin (APC)-conjugated CD172α (SIRPA, cat. no. 17-1721-82) Monoclonal Antibody (eBioscience, Thermo Fisher Scientific, San Diego, CA, USA). Labeled samples were measured using the BD FACSCanto™ II flow cytometer and BD FACSDIVA™ software (BD Biosciences, San Jose, CA, USA).

### 4.3. Differentiation Assay

Cardiomyogenic differentiation of AF-MSCs was induced using different biomolecules: angiotensin II, retinoic acid, EGCG (epigallocatechin gallate), and vitamin C as well as their combinations. All agents were purchased from Sigma-Aldrich Chemie GmbH, Taufkirchen, Germany. Differentiation conditions are provided in Table 1. Several concentrations of these agents were tested, and the optimal ones were chosen for the induction of differentiation (Figure S1, Supplementary Files). The differentiation medium for all agents was DMEM with low glucose (1 g/L), 10% fetal bovine serum (FBS), 100 U/mL penicillin, and 100 µg/mL streptomycin (Gibco, Thermo Fisher Scientific, New York, NY, USA). The differentiation time was 12 days, i.e., the minimum time required to achieve evidence of initiated differentiation according to our previous publication [7]. Each cell population was differentiated in three replicates, and undifferentiated AF-MSCs were used as a control. 

### 4.4. RNA Isolation and Gene Expression Analysis Using RT-qPCR 

For isolation of total RNA from undifferentiated and differentiated cells, TRIzol^®^ reagent (Thermo Fisher Scientific, San Diego, CA, USA) was used. For the gene expression analysis, complementary DNA (cDNA) was synthesized with the “Maxima First Strand cDNA Synthesis Kit for RT-qPCR” (Thermo Fisher Scientific, Vilnius, Lithuania). RT-qPCR was performed with “Maxima SYBR Green qPCR Master Mix” (Thermo Fisher Scientific, Vilnius, Lithuania) and Rotor-Gene 6000 thermal cycler (QIAGEN Instruments AG, Hombrechtikon, Switzerland). The relative gene expression was calculated using the ∆∆Ct method (compared to undifferentiated control), while the GAPDH gene was used for normalization of the messenger RNA (mRNA) amount. The list of used primers (Metabion International AG, Planegg-Steinkirchen, Germany) is provided in the Supplementary Methods (see Table S1, Supplementary Files).

### 4.5. Total Protein Isolation and Western Blot Analysis

Total proteins from control and differentiated AF-MSCs were isolated following the protocol described in Glemžaitė and Navakauskienė [53]. Adult mouse heart, washed with phosphate-buffered saline (PBS), was homogenized in liquid nitrogen and lysed using 2× SDS lysis buffer. Cells and mouse heart protein lysates were separated in 7.5–15% gradient SDS/PAGE gel, before being transferred onto a PVDF membrane, and target proteins were detected using antibodies against human and mouse H3K4me3 (cat. no. 07-473), H3K9Ac (cat. no. 07-352), H4hyperAc (cat. no. 06-946), H3K27me3 (cat. no. 17-622) (Millipore, Burlington, MA, USA), Connexin 43 (cat. No. 13-8300; Thermo Scientific, Bannockburn, IL, USA), and Nkx2.5 (cat. No. GTX105711; GeneTex, Irvine, CA, USA). Antibodies against GAPDH (cat. no. ab9485; Abcam, Cambridge, UK) or histone H3 (Millipore, Burlington, MA, USA) were used as a protein loading control. Secondary antibodies against mouse, rabbit, and goat antibodies (DAKO, Glostrup, Denmark) were conjugated with horseradish peroxidase, and enhanced chemiluminescence was detected using Clarity^TM^ Western ECL Substrate and a ChemiDoc^TM^ XRS+ system with Image Lab^TM^ Software (Bio-Rad Laboratories, Irvine, CA, USA). The relative density of each band was evaluated using Image J software (NIH, Bethesda, MD, USA) and normalized to the GAPDH or H3 loading control. 

### 4.6. Immunofluorescence

Control and differentiated AF-MSCs were seeded on the coverslips, fixed with 4% paraformaldehyde solution in 1× PBS, permeabilized with 10% Triton X-100/PBS, and blocked with 1% BSA/10% goat serum/PBS. All procedures were performed according to the protocol provided in Gasiūnienė et al. [7]. Connexin 43 was detected using primary rabbit against Connexin 43 and secondary goat anti-rabbit IgG (H + L) Highly Cross-Adsorbed, Alexa Fluor-488 antibodies (Thermo Fisher Scientific, Bannockburn, IL, USA). F-actin was labeled with Alexa Fluor-594 Phalloidin (Thermo Fisher Scientific, Bannockburn, IL, USA). Nkx2.5 was detected using primary rabbit against Nkx2.5 and secondary goat anti-rabbit IgG (H + L) Highly Cross-Adsorbed, Alexa Fluor-594 antibodies (Thermo Fisher Scientific, Bannockburn, IL, USA). F-actin in these samples was labeled with Alexa Fluor-488 Phalloidin (Thermo Fisher Scientific, Bannockburn, IL, USA). Nuclei were stained with 300 nM DAPI (Invitrogen, Hillsboro, OR, USA). Coverslips were mounted using Dako Fluorescent Mounting Medium (Agilent Technologies, Santa Clara, CA, USA), and the localization of Connexin 43 and Nkx2.5 was analyzed using a Zeiss Axio Observer (Oberkochen, Germany) fluorescent microscope, with a 63× immersion objective and Zen BLUE software.

### 4.7. Extracellular Flux Analysis

The energetic profile of differentiated and control cells was determined using the Seahorse XFp Extracellular Flux Analyzer and Cell Energy Phenotype Test Kit (Agilent Technologies, Santa Clara, CA, USA). Using this analyzer, we simultaneously measured oxygen consumption rate (OCR) and extracellular acidification rate (ECAR), firstly in baseline conditions (without inhibitors of the electron transfer chain, oligomycin and FCCP) and then in induced stress conditions (after the addition of the above-mentioned inhibitors). After the analysis, cells were collected for normalization. They were lysed using RIPA buffer (150 mM NaCl, 10 mM EDTA, pH 8.0, 10 mM Tris, pH 7.4, 0.1% SDS, 1% deoxycholate, 1% NP-40 in PBS, pH 7.6). Total protein concentrations were determined using the DC Protein Assay (BioRad Laboratories, Irvine, CA, USA) and spectrophotometer Infinite M200 Pro (Tecan, Switzerland). Then, OCR and ECAR values were normalized to the total amount of protein in each well and expressed per µg of protein. The calculated parameters involved (a) the OCR/ECAR ratio, determined from the normalized OCR and normalized ECAR, (b) the metabolic potential, i.e., the percentage increase of stressed OCR over baseline OCR and stressed ECAR over baseline ECAR, and (c) the cell energy phenotype, determined from OCR and ECAR values at baseline and in induced stress conditions. More details are provided in the Supplementary Methods.

### 4.8. Statistical Analysis

All experiments were performed in triplicate; data were expressed as the mean ± SD. Statistical analysis was done using one-way ANOVA with Tukey post hoc test in GraphPad Prism software (San Diego, CA, USA).

## 5. Conclusions

Biologically active compounds, namely, angiotensin II, retinoic acid, EGCG, vitamin C, and their combinations with angiotensin II, are able to induce alterations in AF-MSCs at the phenotypic, genetic, protein, metabolic, and epigenetic levels to a different extent leading to the formation of cardiomyocyte progenitors that may become functional heart cells upon maturation in vitro or in vivo.

## Figures and Tables

**Table 1:** Differentiation inducers and conditions.

Inducer and Its Abbreviation	Working Concentration	Stock Solution
Angiotensin II (AngII)	0.1 µM	1 mM in distilled water
Retinoic acid (RA)	1 µM	2 mM in Dimethyl sulfoxide (DMSO)
Epigallocatechin-3-gallate (EGCG)	30 µM	20 mM in DMSO
Vitamin C (Vit. C)	50 µg/mL	50 mg/mL in distilled water
Angiotensin II with retinoic acid (AngII + RA)	0.1 µM AngII + 1 µM RA	-
Angiotensin II with epigallocatechin gallate (AngII + EGCG)	0.1 µM AngII + 30 µM EGCG	-
Angiotensin II with Vitamin C (AngII + Vit. C)	0.1 µM AngII + 50 µg/mL vitamin C	-

## Supplementary Materials

Supplementary materials can be found at www.mdpi.com/1422-0067/21/22/8752.
