# Supplementary material for: The Effect of Angiotensin II, Retinoic Acid, EGCG, and Vitamin C on the Cardiomyogenic Differentiation Induction of Human Amniotic Fluid-Derived Mesenchymal Stem Cells
Source: Int J Mol Sci. 2020 Nov 19;21(22):8752. doi: 10.3390/ijms21228752 (PMC7699548; doi:10.3390/ijms21228752)
Supplement: Supplementary file 1 [file ijms-21-08752-s001.zip › Supplementary Figure S1.pdf]

Supplementary Figure S1.

Differentiation Inducer	Tested Concentrations	References*
Angiotensin II	0,1 µM	Xing et al., 2012<br>Gasiūnienė et al., 2019
RA	1 µM	Wobus et al., 1994
	3 µM	Wobus et al., 1997
EGCG	10 µM	Borutinskaitė et al., 2017
	30 µM	Vitkevičienė et al., 2018
Vitamin C	25 µg/ml<br>50 µg/ml<br>75 µg/ml	Cao et al., 2012; Talkhabi et al., 2015; Abbey et al., 2017; Fujisawa et al., 2018; Liu et al., 2019

* Full references provided in the page 3

(A)

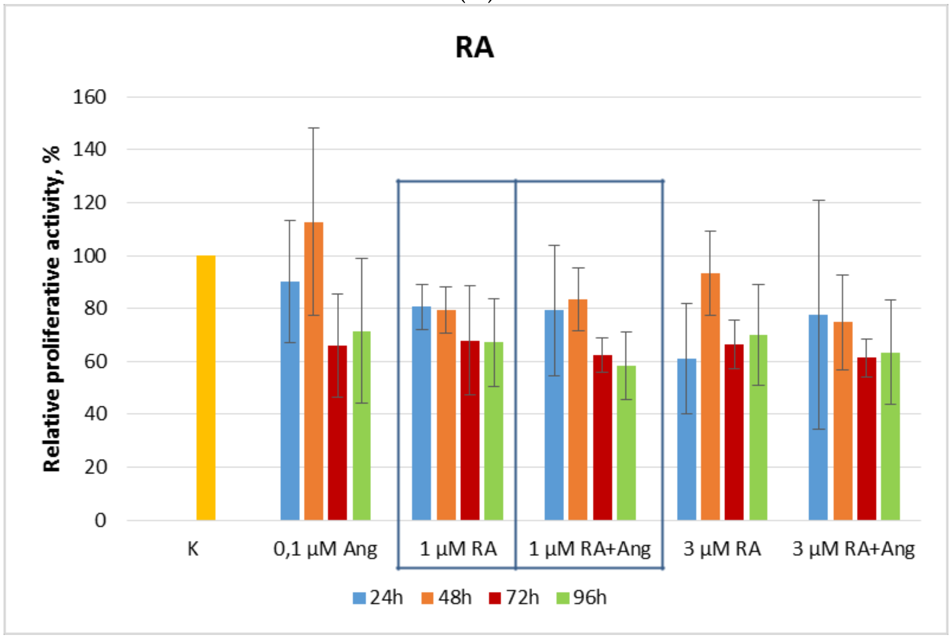

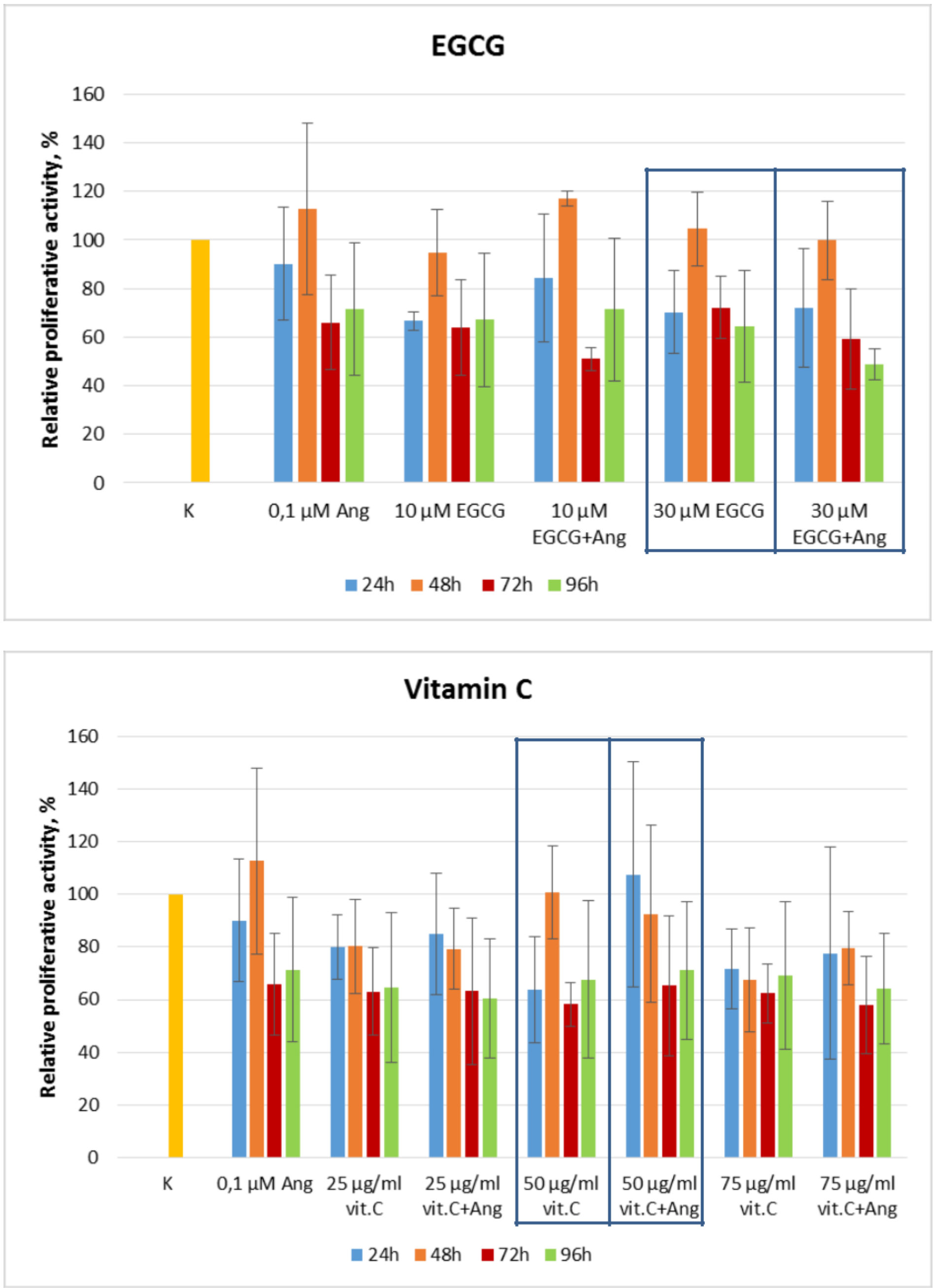

(**B**)

Figure S1. Determination of concentrations of Angiotensin II (AngII), retinoic acid (RA), EGCG and vitamin C (Vit. C) used for cardiomyogenic differentiation induction of AF-MSCs. (**A**) The sources of applied concentrations of these agents. (**B**) MTT data showing the relative proliferation activity of AF-MSCs using different concentrations of differentiation inducers. Data are presented as mean ± SD (*n* = 3).

References

1. Xing Y, Lv A, Wang L, Yan X. The combination of angiotensin II and 5-azacytidine promotes cardiomyocyte differentiation of rat bone marrow mesenchymal stem cells. Mol Cell Biochem. 2012 Jan;360(1-2):279-87. doi: 10.1007/s11010-011-1067-z.

2. Gasiūnienė M, Petkus G, Matuzevičius D, Navakauskas D, Navakauskienė R. Angiotensin II and TGF-β1 induce alterations in human amniotic fluid-derived mesenchymal stem cells leading to cardiomyogenic differentiation initiation. Int J Stem Cells. 2019a Apr 30. doi:10.15283/ijsc18126.

3. Wobus AM, Rohwedel J, Maltsev V, Hescheler J. In vitro differentiation of embryonic stem cells into cardiomyocytes or skeletal muscle cells is specifically modulated by retinoic acid. Roux Arch Dev Biol. 1994 Oct;204(1):36-45. doi: 10.1007/BF00189066.

4. Wobus AM, Kaomei G, Shan J, Wellner MC, Rohwedel J, Ji Guanju, Fleischmann B, Katus HA, Hescheler J, Franz WM. Retinoic acid accelerates embryonic stem cell-derived cardiac differentiation and enhances development of ventricular cardiomyocytes. J Mol Cell Cardiol. 1997 Jun;29(6):1525-39.

5. Borutinskaitė V, Virkšaitė A, Gudelytė G, Navakauskienė R. Green tea polyphenol EGCG causes anti-cancerous epigenetic modulations in acute promyelocytic leukemia cells. Leuk Lymphoma. 2018 Feb;59(2):469-478. doi: 10.1080/10428194.2017.1339881.

6. Vitkeviciene A, Baksiene S, Borutinskaite V, Navakauskiene R. Epigallocatechin-3-gallate and BIX-01294 have different impact on epigenetics and senescence modulation in acute and chronic myeloid leukemia cells. Eur J Pharmacol. 2018 Nov 5;838:32-40. doi:1016/j.ejphar.2018.09.005.

7. Cao N, Liu Z, Chen Z, Wang J, Chen T, Zhao X, Ma Y, Qin L, Kang J, Wei B, Wang L, Jin, Y., Yang HT. Ascorbic acid enhances the cardiac differentiation of induced pluripotent stem cells through promoting the proliferation of cardiac progenitor cells. Cell Res. 2012 Jan;22(1):219-36. doi: 10.1038/cr.2011.195.

8. Talkhabi M, Pahlavan S, Aghdami N, Baharvand H. Ascorbic acid promotes the direct conversion of mouse fibroblasts into beating cardiomyocytes. Biochem Biophys Res Commun. 2015 Aug 7;463(4):699-705. doi: 10.1016/j.bbrc.2015.05.127.

9. Abbey D, Seshagiri PB. Ascorbic acid-mediated enhanced cardiomyocyte differentiation of mouse ES-cells involves interplay of DNA methylation and multiple-signals. Differentiation. 2017 Jul - Aug;96:1-14. doi: 10.1016/j.diff.2017.04.001.

10. Fujisawa K, Hara K, Takami T, Okada S, Matsumoto T, Yamamoto N, Sakaida I. Evaluation of the effects of ascorbic acid on metabolism of human mesenchymal stem cells. Stem Cell Res Ther. 2018 Apr 6;9(1):93. doi: 10.1186/s13287-018-0825-1.

11. Liu W, Liu H, Wang Y, Zhang L, Wang C, Li H. Ascorbic acid induces cardiac differentiation of white adipose tissue-derived stem cells. Mol Cell Biochem. 2019 Jan;450(1-2):65-73. doi: 10.1007/s11010-018-3373-1.
