# Supplementary material for: The Effect of Angiotensin II, Retinoic Acid, EGCG, and Vitamin C on the Cardiomyogenic Differentiation Induction of Human Amniotic Fluid-Derived Mesenchymal Stem Cells
Source: Int J Mol Sci. 2020 Nov 19;21(22):8752. doi: 10.3390/ijms21228752 (PMC7699548; doi:10.3390/ijms21228752)
Supplement: Supplementary file 1 [file ijms-21-08752-s001.zip › Supplementary Table S1.pdf]

Supplementary Table S1. Primers used in RT-qPCR

Gene	Primer Sequence (5′-3′)	Product Size (bp)
SOX2	Forward TGGACAGTTACGCGCACAT<br>Reverse CGAGTAGGACATGCTGTAGGT	215
REX1	Forward GCCTTATGTGATGGCTATGTGT<br>Reverse ACCCCTTATGACGCATTCTATGT	96
OCT4	Forward CGAGAAGGATGTGGTCCGAG<br>Reverse CAGAGGAAAGGACACTGGTC	136
NANOG	Forward AGATGCCTCACACGGAGACT<br>Reverse GTTTGCCTTTGGGACTGGTG	96
TNNT2	Forward CATGGAGAAGGACCTGAATGA<br>Reverse CGTCTCTCGATCCTGTCTTTG	108
MYH6	Forward CCACCCAAGTTCGACAAGAT<br>Reverse CACAGAAGAGGCCCGAGTAG	127
NKX2-5	Forward CAAGTGTGCGTCTGCCTTT<br>Reverse GCGCACAGCTCTTTCTTTTC	105
DES	Forward CTGAGCAAAGGGGTTCTGAG<br>Reverse ACTTCATGCTGCTGCTGTGT	109
GAPDH	Forward AGTCCCTGCCACACTCAG<br>Reverse TACTTTATTGATGGTACATGACAAGG	123
SCN5A	Forward TCATCGTAGACGTCTCTCTGGT<br>Reverse GGCTCTTGTTGTTCACGATGGT	318
CACNA1D	Forward GGGCAATGGGACCTCATAAATAA<br>Reverse TTACCTGGTTGCGAGTGCATTA	141
KCNJ12	Forward GCCAGCTAGGCTCTGTTTGTG<br>Reverse CTGAGACACATCTCTAAGGTAC	152
KCND3	Forward AGAGAGCTGATAAACGCAGGG<br>Reverse CAGGCAGTGCAGCAGGTGAT	209
HCN2	Forward CGCCTGATCCGCTACATCCAT<br>Reverse AGTGCGAAGGAGTACAGTTCACT	230
PPARGC1A	Forward GCAATTGAAGAGCGCCGTGTGA<br>Reverse CTGTCTCCATCATCCCGCAGAT	134
NRF1	Forward AGGCTGGGGGAAAGAAAG<br>Reverse CCAACCTGGATAAGTGAGAC	303
HIF1A	Forward CCAACAGTAACCAACCTCAG<br>Reverse TCCTGTGGTGACTTGTCCTT	302

Extracellular Flux analysis

Extracellular Flux analysis was done using Seahorse XFp Extracellular Flux analyzer, Cell Mito Stress Test Kit and Cell Energy Phenotype Test Kit (Agilent Technologies, USA). The device simultaneously detects changes in oxygen concentration and in proton concentration (or pH) in the assay medium.

<u>Cell Energy Phenotype Test Kit</u> measures the basal oxygen consumption rate (OCR) and then OCR after the injection of the stressor mix (1 μM of Oligomycin and 1 μM of FCCP). Oligomycin inhibits ATP production by the mitochondria and causes a compensatory increase in the rate of glycolysis as the cells attempt to meet their energy demands via the glycolytic pathway. FCCP depolarizes the mitochondrial membrane and drives OCR higher as the mitochondria attempt to restore the mitochondrial membrane potential. The main parameters are as follows:

- Oxygen consumption rate (OCR): The rate of decrease of oxygen concentration in the assay medium. OCR is a measure of the rate of mitochondrial respiration of the cells.
- Extracellular acidification rate (ECAR): The rate of increase in proton concentration (or decrease in pH) in the assay medium. ECAR is a measure of the rate of glycolysis of the cells.
- Baseline phenotype: OCR and ECAR of cells at starting assay conditions.

- Stressed phenotype: OCR and ECAR of cells in the presence of stressor compounds.
- Metabolic potential: Percentage increase of stressed OCR over baseline OCR and stressed ECAR over baseline ECAR. Metabolic potential is the measure of cells' ability to meet the energy demand via respiration and glycolysis.
